# Scientific Items

**Published:** 1882-04-20

**Authors:** 


### SCIENTIFIC ITEMS.

**Locusts as Food.**—Some writer remarked long ago that, in our warfare against injurious insects, it might be well for us to turn the tables upon them by *eating* them. This has been done in the East, so far as locusts are concerned, ever since the days of John the Baptist, and probably from a much earlier period. A recent writer says: "In Arabia and Northern Africa, locusts are still used as food, and form an article of commerce. The inhabitants usually tear off their wings and wing coverts, and then bake them. *Gryllus lineolc* (Fabr.) seems to be the species which is eaten and prepared in the manner above detailed, in Barbary. The natives of Senegal dry another species, of which the body is yellow, spotted with black, and which Shaw and Denon have figured in the account of their voyage in Africa; they then reduce them to powder, which they use as flour. Captain Burton tells us that the black, leather-like variety of locust, called by the Arabs, 'Satan's ass,' is eaten by the Africans, as are many other edibles upon which strangers look with disgust.

"There is some compensation in the circumstance that if the locusts devour the food of man they are themselves a source of food. In Morocco they are collected in sacks by night, and first boiled in salt and water, and then fried. Only the softer part of the body is eaten, much as we eat prawns, which they resemble in taste. They are considered to be wholesome food, and in perfection as soon as the insects can fly."—*Journal of Chemistry.*

**The Wonders of Paper.**—At the Melbourne Exhibition, held recently, there was a complete dwelling house made entirely of paper, and furnished throughout with the same material. There were paper walls, paper roofs, paper ceilings, paper floorings, paper joists, paper stairways, paper carpets, paper bedding, paper chairs, paper sofas, paper lamp, paper frying-pans, and even the stoves, in which bright fires were kept burning daily, were of *papier mache*, and when the fabricator of this mansion gave a banquet in this dwelling; the table-cloths, the napkins, the plates and cups and saucers, the bottles and the tumblers, and even the knives and forks, were likewise made of paper.—*Journal of Chemistry.*

**Canned Meats and Vegetables.**—Otto Hener, F. C. S., publishes in the Lancet the results of his experiments, both chemical and physiological, upon the occasional injurious effects of canned goods. He says that tin, even when perfectly pure, is far more readily attacked by food matters than is commonly supposed; it is to be found in comparatively large amounts in the overwhelming majority of canned goods, irrespective of the nature of the same. Acid fruits, such as peaches and cherries, corrode the tin to an appalling extent; but even meats, nay, condensed milk, dissolve and become contaminated with serious quantities of the metal. A can of soup examined contained 35 milligrams (one-half grain) of tin to the pound, and one of condensed milk 8 mg. (one-eighth grain) and of oysters 45 mg. (0.7 grain) to the pound.

Carbonic acid water attacks tin, and in all samples of soda-water, and other acetated beverages tested, tin was found. The question then arises, Is tin, when taken into the system, injurious to health, or not? This seems to be as yet an unsettled question, but Hehner's experiments on pigs go to show that, while *stannic* compounds are not injurious, tin in a *stannous* condition is a virulent irritant poison, and it is in the latter condition that tin dissolves in the animal or vegetable substances used for food.—*Ibid.*

**Artificial Leather.**—Among the recent patents granted at Washington is one for artificial leather, which is a plastic compound for coating articles to imitate leather, etc., consisting of glue, mastic, dextrine, glycerine, chloride of iron, chrome-alum and a suitable pigment. It is said to wear nearly as well as leather, and costs but half as much. It is used in the manufacture of ladies' fine boots and slippers, and by carriage-makers, book-binders, and upholsterers.—*Ibid.*

**Electrical Experiment.**—The following simple electrical experiment is described in L'Electricien: A small box of pasteboard is closed with lid of fine glass, on the upper surface of which collodion is applied several times (but not so much as to render the lid opaque). In the box are placed insect forms, made of sponge or cotton. On rubbing the collodion surface with dry fingers in dry weather, the insects move about in a curious manner. *Ibid.*

**Prof. Edward C. Pickering,** of Harvard College, says that in undertaking to measure the intensity of the light of the satellites of Mars, he had occasion to need an extremely small hole. A hole about the twenty-five hundreth part of an inch in diameter was finally secured.—*Philo. Jour.*

**M. Pasteur** has resolved to continue his researches into the means of preventing disease by destroying or nullifying the virulence of the germs, and is about to visit Bordeaux lazaretto with the view of studying yellow fever, which he hopes, to conquer by means of inoculation.—*Ibid.*

**The experiments** of a famous Swedish chemist, prolonged over two years, makes it definitely certain that separating cream by the centrifugal secures 10 per cent. more of it than any other process, while if the cream is at once churned what chemist and other experimenters pronounce the best lasting and best keeping butter is obtained; the refuse—the skimed milk and buttermilk—are sweet—that is, in their most valuable condition—and the milk has been in the course of a few hours turned into money. This appears to be the ultimate perfection of scientific buttermaking.—*Ibid.*

